# The Detection of *Yr* Genes in Xinjiang Wheat Cultivars Using Different Molecular Markers

**DOI:** 10.3390/ijms241713372

**Published:** 2023-08-29

**Authors:** Minghao Zhang, Ainisai Saimi, Qi Liu, Zeyu Ma, Jing Chen

**Affiliations:** 1 Key Laboratory of the Pest Monitoring and Safety Control of Crops and Forests of the Xinjiang Uygur Autonomous Region, College of Agronomy, Xinjiang Agricultural University, Urumqi 830052, China; 17853435690@163.com (M.Z.); anisa1010@163.com (A.S.); mzynswy@163.com (Z.M.); chenj@xjau.edu.cn (J.C.); 2 Key Laboratory of Prevention and Control of Invasive Alien Species in Agriculture & Forestry of the North-Western Desert Oasis, Ministry of Agriculture and Rural Affairs, Urumqi 830052, China

**Keywords:** resistance breeding, resistance gene detection, wheat stripe rust, Xinjiang wheat cultivars

## Abstract

Wheat stripe rust is a fungal disease caused by *Puccinia striiformis* f. sp. *Tritici* (*Pst*). It significantly impacts wheat yields in Xinjiang, China. Breeding and promoting disease-resistant cultivars carrying disease-resistance genes remains the most cost-effective strategy with which to control the disease. In this study, 17 molecular markers were used to identify *Yr5*, *Yr9*, *Yr10*, *Yr15*, *Yr17*, *Yr18*, *Yr26*, *Yr41*, *Yr44*, and *Yr50* in 82 wheat cultivars from Xinjiang. According to the differences in SNP loci, the KASP markers for *Yr30*, *Yr52*, *Yr78*, *Yr80*, and *Yr81* were designed and detected in the same set of 82 wheat cultivars. The results showed that there was a diverse distribution of *Yr* genes across all wheat cultivars in Xinjiang, and the detection rates of *Yr5*, *Yr15*, *Yr17*, *Yr26*, *Yr41*, and *Yr50* were the highest, ranging from 74.39% to 98.78%. In addition, *Yr5* and *Yr15* were prevalent in spring wheat cultivars, with detection rates of 100% and 97.56%, respectively. A substantial 85.37% of wheat cultivars carried at least six or more different combinations of *Yr* genes. The cultivar Xindong No.15 exhibited the remarkable presence of 11 targeted *Yr* genes. The pedigree analysis results showed that 33.33% of Xinjiang wheat cultivars shared similar parentage, potentially leading to a loss of resistance against *Pst*. The results clarified the *Yr* gene distribution of the Xinjiang wheat cultivars and screened out varieties with a high resistance against *Pst*.

## 1. Introduction

Wheat stripe rust is one of the most serious crop diseases threatening wheat production, and it significantly reduces wheat yield and quality [1]. It has the characteristics of high epidemic frequency, wide occurrence range, and regional prevalence [2]. China is an important epidemic region for stripe rust in the world, and it may lead to large economic losses when the disease is severe [3]. Destructive epidemics in China in 1950, 1964, 1990, 2002, and 2017 caused yield losses exceeding 6.0, 3.2, 1.8, 1.3, and 1.5 million metric tons, respectively [4]. Due to its unique geographical barriers, climatic conditions, and wheat growing environment, Xinjiang was classified as a relatively independent epidemiological zone for wheat stripe rust in China [5]. In Xinjiang, wheat is distributed in all wheat growing areas except the Turpan region. The Yili Kazak Autonomous Prefecture and Kashgar Prefecture have the largest wheat planting areas [6]. As a prevalent wheat disease in Xinjiang, wheat stripe rust has emerged as a pivotal factor constraining local wheat production [7]. There are many ways to control wheat stripe rust, but breeding and promoting varieties carrying resistance genes is currently the most cost-effective and environmentally friendly method [8]. According to the references, more than 100 *Yr* genes have been detected in wheat, and 84 of them have received permanent nomenclature. More than 70% of the 84 *Yr* genes were all-stage resistance (ASR) genes, and the rest were adult-phase resistance (APR) genes [9]. Wild species and common species of wheat are the main sources of these disease-resistance genes. Due to high disease resistance and stable inheritance, resistance genes such as *Yr9*, *Yr10*, *Yr17*, and *Yr24*/*Yr26* were extensively integrated into wheat cultivar breeding. However, high genetic variation in the pathogen population and the rapid rate of selection for new virulent species lead to some *Yr* genes losing their resistance. Therefore, the identification of *Yr* gene distribution and *Yr* gene combinations would be useful for developing new wheat cultivars for the sustainable control of stripe rust.

With the development of research in molecular biology, more and more molecular biology techniques have been applied to study wheat stripe rust resistance genes, such as simple sequence repeats (SSRs), sequence tagged sites (STSs), and competitive allele-specific polymerase chain reactions (KASP). KASP, an advanced, high-throughput genotyping technology, emerged as a novel molecular marker technology based on single-nucleotide polymorphisms (SNPs) and insertion deletions (InDels). Due to economic efficiency and heightened effectiveness, KASP has been widely used in molecular-assisted breeding [10]. At present, more than 100 functional markers (FMs) have been developed for the detection of important function genes in wheat [11]. With the development of wheat genome sequencing, FMs could increase rapidly, which could be translated into more molecular KASP markers to improve the efficiency of wheat breeding. A total of 124 KASP markers were used to detect the distribution of beneficial alleles in 213 wheat cultivars [12]. The 7BL QTL was developed and identified as a new gene, and was permanently designated as a KASP marker of *Yr79* [13]. The *Yr* genes were evaluated in a core collection of 305 Chinese wheat cultivars; *Yr9*, *Yr10*, *Yr17*, *Yr18*, *Yr26*, *Yr30*, *Yr41*, *Yr78*, and *Yr80* were detected, at different frequencies, in the collection [14]. 

In this study, the disease-resistance genes *Yr5*, *Yr9*, *Yr10*, *Yr15*, *Yr17*, *Yr18*, *Yr26*, *Yr41*, *Yr44*, and *Yr50* were detected using the different molecular markers in spring and winter wheat cultivars in Xinjiang, China. Through the differences in SNP loci, KASP primers were also designed to detect the *Yr30*, *Yr52*, *Yr78*, *Yr80*, and *Yr81* genes. The results clarified the distribution of resistance genes against stripe rust in Xinjiang wheat cultivars and provided a scientific basis for wheat disease resistance breeding and the rational distribution of wheat cultivars in Xinjiang.

## 2. Results

### 2.1. The Detection Results of Yr Genes

**Figure 1:**
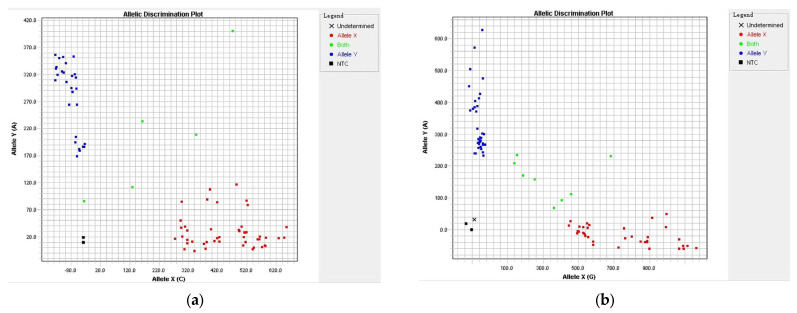
The detection results of *Yr78* (**a**) and *Yr80* (**b**) with KASP markers.

**Figure 2:**
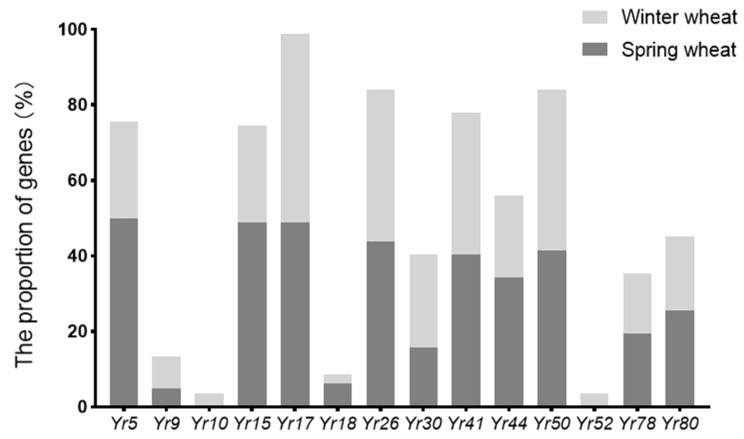
The proportion of *Yr* genes in winter and spring wheat cultivars in Xinjiang.

In this study, 22 developed molecular markers were used to detect 15 *Yr* genes (including *Yr5*, *Yr9*, *Yr10*, *Yr15*, *Yr17*, *Yr18*, *Yr26*, *Yr30*, *Yr41*, *Yr44*, *Yr50*, *Yr52*, *Yr78*, *Yr80*, and *Yr81*) in spring and winter wheat cultivars in Xinjiang (Figure 1a,b and Table 1). The results showed that the detection rates of *Yr5*, *Yr9*, *Yr10*, *Yr15*, *Yr17*, *Yr18*, *Yr26*, *Yr30*, *Yr41*, *Yr44*, *Yr50*, *Yr52*, *Yr78*, *Yr80*, and *Yr81* were 75.61%, 13.41%, 3.66%, 74.39%, 98.78%, 8.54%, 84.15%, 40.24%, 78.05%, 56.1%, 84.15%, 3.66%, 35.37%, 45.12%, and 0, respectively (Figure 2). Among them, *Yr5*, *Yr15*, *Yr17*, *Yr26*, *Yr41*, and *Yr50* had higher detection rates, in the range of 74.39–98.78%. The detection rate of *Yr81* was 0. The detection rates of *Yr5* and *Yr15* in spring wheat cultivars were higher than in winter wheat, standing at 100% and 97.56%, respectively. In order to verify the accuracy of the detection results, all of the genes were sequenced, and the sequencing results were consistent with the detection results. 

### 2.2. The Combination Results of Yr Genes in Wheat Cultivars

**Figure 3:**
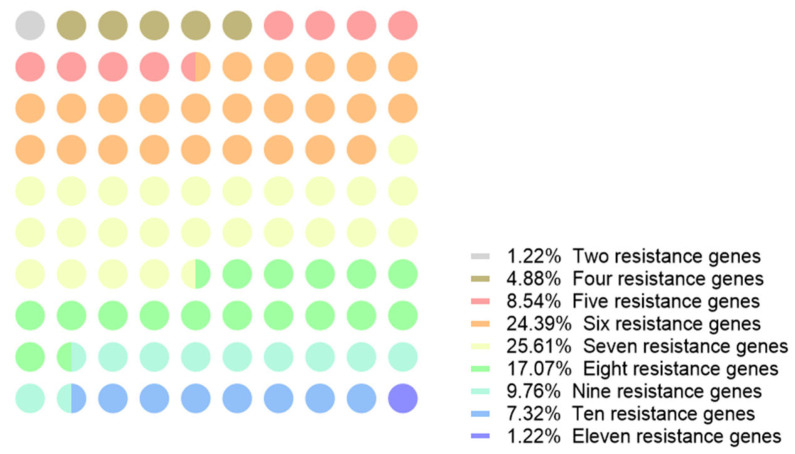
The proportion of different numbers of *Yr* genes in 82 Xinjiang wheat cultivars.

In this study, the results of *Yr* gene detection numbers in different wheat cultivars were as follows: most of the wheat cultivars contained seven *Yr* genes, accounting for 25.61% of all the wheat cultivars; those with six *Yr* genes accounted for 24.39%; and those with eight *Yr* genes accounted for 17.07%. In the cultivar Xindong No.15, the greatest number of *Yr* genes was detected; it had 11 *Yr* genes. In the cultivar Xindong No.49, only *Yr17* and *Yr78* were detected via molecular markers (Figure 3). In the field resistance test, the cultivar Xindong No.49 showed a high susceptibility to *Pst*, with only two *Yr* genes detected. The cultivar Xindong No.15 exhibited robust resistance to *Pst*, with 11 *Yr* genes detected. The more *Yr* genes a wheat cultivar contains, the greater its resistance against *Pst*. While diverse wheat cultivars exhibited distinct combinations of *Yr* genes, the recurrent trend included random amalgamations of five to seven genes, particularly involving *Yr5*, *Yr15*, *Yr17*, *Yr26*, *Yr41*, *Yr44*, and *Yr50*. The distribution frequencies of different *Yr* genes showed great difference, and the variety of gene combinations was comparatively limited.

### 2.3. The Results of Pedigree Analysis

The pedigree information of 66 wheat cultivars was obtained through the cultivar certification inquiry (Table 2). In the all cultivars with known pedigree information, 87.88% were cultivated in the Xinjiang Academy of Agricultural Sciences, Xinjiang Agricultural University and Shihezi University, and 12.12% were introduced from other regions. The 22 varieties were cultivated using the same series of spring and winter wheat as parents, which may be the main reason for the low diversity in *Yr* gene combinations in wheat cultivars in Xinjiang. Xinchun No.2 was the parent of Xinchun No.11. In this study, Xinchun No.11 was detected to have nine shared genes in Xinchun No.2, except *Yr78*. *Yr78* was presumed to be inherited from the wheat of 86-7. We detected eleven *Yr* genes in Xindong No.15, eight of which were the same as those in Xindong No.2. The other genes, *Yr5*, *Yr9*, *Yr44*, and *Yr78*, were presumed to be inherited from Zhongyin No.5. Because Xinchun No.11 and Xindong No.15 carried more *Yr* genes, their disease resistance to *Pst* should be better than that of their parents, Xinchun No.2 and Xindong No.2, which had been verified in the previous field study [15].

## 3. Discussion

The strategy of using combining *Yr* genes to improve wheat resistance against *Pst* has become a well-established practice in breeding. In this study, a total of 22 molecular markers were used to detect 15 *Yr* genes in 82 wheat cultivars in Xinjiang. The results showed that there were a large number of *Yr* genes in the 82 wheat cultivars, but they also had a great difference in detection rate. The variety of *Yr* gene combinations was relatively simplified; we mainly found *Yr5*, *Yr15*, *Yr17*, *Yr26*, *Yr41*, *Yr44*, and *Yr50*. Pedigree analysis illuminated a significant trend: the majority of these wheat cultivars shared common parents, originating from the same local parental cultivars, which meant that the Xinjiang wheat cultivars had a homogenous background. When the *Pst* evolves into new virulent races that can overcome *Yr* genes, wheat stripe rust could cause an outbreak and pandemic, yielding large economic losses. Therefore, the rational utilization of *Yr* gene combinations is very necessary for disease resistance, which can enhance the genetic diversity within the wheat population and control wheat stripe rust for long-term effectiveness.

ASR genes are *Yr* genes that are resistant to *Pst* in all wheat growth stages [16]. High-temperature adult plant (HTAP) resistance genes are also widely used in breeding because of their good resistance performance. Currently, the combination of ASR and HTAP genes is a conventional strategy in breeding, and it has been demonstrated in breeding practices in the United States [17]. In this study, *Yr9* was used as the ASR gene and *Yr18* as the HTAP gene. The *Yr9* gene was from a wheat–rye 1BL/1RS translocation line; its derivatives played an important role in wheat disease resistance breeding, but due to the virulence performance of CYR29, *Yr9* lost its resistance. Only 11 samples of this gene were detected in this study. The detection rate of *Yr9* accounted for 13.41% of the total. Additionally, Han et al. reported that the combination of *Yr9* and other *Yr* genes for breeding could enhance the disease resistance of wheat varieties [18]. Therefore, the rational use of combination *Yr* genes in wheat breeding should be considered in future. The *Yr18* gene encodes an ATP-binding cassette (ABC) transporter that is resistant to multiple diseases, such as stripe rust, leaf rust, stem rust, powdery mildew, and leaf blight [19]. Nevertheless, the detection rate of this gene was low in this study. The detection rate of *Yr18* accounted for 8.54% of the total. Therefore, it could be preferentially used for breeding in combination with ASR genes such as *Yr9* for disease resistance in breeding practices in Xinjiang.

The disease resistance of a new wheat variety was determined using the pedigree information of the wheat parents. In this study, 87.88% of the wheat varieties were cultivated locally, and 33.33% of them shared a similar parentage, which may be the main reason for their resistance loss against *Pst*. The Xinchun No.2 and Xindong No.2 varieties were the most frequently used parents in local Xinjiang wheat breeding. In the future breeding work, high-quality resistance introduction and different *Yr* gene combinations will be better strategies that will provide more selection for resistance improvement and resistance breeding. 

Nowadays, the most common international breeding methods include conventional breeding, molecular marker-assisted selection breeding, and transgenic breeding. Molecular marker-assisted selection breeding and transgenic breeding have emerged as superior approaches, enabling the targeted enhancement of specific traits with remarkable efficiency. Due to product commercialization, transgenic breeding has a more limited use than molecular marker-assisted selection breeding [20]. With the development of high-throughput sequencing technology, a third generation of molecular markers has been developed; the first generation was based on molecular hybridization technology, whereas the second generation was based on polymerase chain reaction (PCR) technology. Single-nucleotide-polymorphism-based molecular markers are known as the third generation [21]. The powdery mildew resistance gene in wheat, *PmCH7087*, was located using the KASP marker within the interval of 9.68 Mb [22]. The wheat stripe rust resistance gene *Yr26* was precisely localized using KASP markers WRS270 and WRS290 [23]. In this study, the second- and third-generation molecular marker techniques were used to detect the disease-resistance genes of Xinjiang wheat cultivars. The results showed that the KASP method had a higher flexibility and lower economic cost than the conventional molecular marker method.

## 4. Materials and Methods

### 4.1. Materials

A total of 82 wheat cultivars from Xinjiang were used in this study, of which 41 were spring wheat varieties and 41 were winter wheat varieties. AVS near-isogenic lines of wheat carrying *Yr5*, *Yr9*, *Yr10*, *Yr15*, *Yr17*, *Yr18*, *Yr26*, *Yr30*, *Yr41*, *Yr44*, *Yr50*, *Yr52*, *Yr78*, *Yr80*, and *Yr81* were used as positive controls for detection. All wheat cultivars were provided by the Laboratory of Plant Disease Epidemiology of Xinjiang Agricultural University. The conventional PCR molecular marker primers used in the study were synthesized by Beijing Bomade Gene Technology Co., Ltd. (Beijing, China), and the KASP primers were synthesized by BGI.

### 4.2. DNA Extraction

The wheat was grown in a phytotron at the Xinjiang Agricultural University. DNA from 20 mg leaves of varieties in the seedling stage was extracted using the cetyl trimethyl ammonium bromide (CTAB) method [24,25]. The DNA concentration was detected using a spectrophotometer; it was diluted to 50 ng/µL by adding 1 × TE. The integrity of the extracted genomic DNA was examined using 1% agarose gel electrophoresis.

### 4.3. PCR Amplification

*Yr5*, *Yr9*, *Yr10*, *Yr15*, *Yr17*, *Yr18*, *Yr26*, *Yr41*, *Yr44*, and *Yr50* genes were detected using the developed molecular markers; sequence primers are shown in Table 3. PCR was performed in a 20 µL reaction mixture containing 1 µL (50 ng/µL) of template DNA, 10 µL of PCR Mix, 1.5 µL of forward primer, 1.5 µL of reverse primer, and 6 µL of ddH_2_O. The amplification procedure was an initial 5 min of denaturation at 94 °C, then 35–40 cycles of 1 min denaturation at 94 °C, 1–2 min of annealing at 45–65 °C, and 1 min extension at 72 °C. Step extension was 10 min at 72 °C and, finally, 10 °C indefinitely. The PCR products were subjected to 1–3% agarose gel electrophoresis (the gel concentration was determined by target fragment size).

The complete coding sequences of wheat stripe rust resistance genes *Yr30*, *Yr52*, *Yr78*, *Yr80*, and *Yr81* were obtained from the published literature and the NCBI website [2,38,39,40,41]. The KASP primers were designed according to the standard KASP guidelines, and the specific KASP primer design sites and sequences are shown in Table 4. Five *Yr* genes were verified by genotyping and sequencing [42]. The PCR amplification system was 5 µL, including 1 µL DNA template, 0.07 µL 72 × Assay Mix, 2.5 µL master mix, and 1.43 µL ddH_2_O. The amplification conditions were an initial 15 min of denaturation at 94 °C for 20 s and annealing at 61 °C for 1 min. Then, each cycle was reduced by 0.6 °C for 10 cycles of denaturation at 94 °C 20 s, then annealed at 55 °C for 1 min for 29 cycles, and lastly maintained at 30 °C for 1 min [43]. The amplified PCR products were fluorescence tested using a SNP typing detector [23]. The quality detection rate of the four genes’ loci was over 90%, except the *Yr52* locus, which had a quality detection rate of 82%. In order to verify the accuracy of the KASP genotyping results, the relevant loci of the KASP genotyping were sequenced.

### 4.4. Pedigree and Data Analysis

The background information of the wheat cultivars was obtained from the authoritative website of the Chinese Seed Industry Data Platform “http://202.127.42.47:6010/SDSite/Home/Index” (accessed on 10th August 2022). Then, the pedigree information and the sources of wheat resistance were obtained. 

The KASP marker genotyping data were exported, visualized, and interpreted using SDS 2.4 software; then, the wheat cultivars with undetectable signals or poor-quality detected signals were excluded [44]. GraphPad Prism 9 was used for statistical analysis.

## 5. Conclusions

The combination of *Yr* genes, both in terms of their amount and type, exhibited a significant and positive correlation with the resistance of wheat cultivars against *Pst*. The more *Yr* genes the wheat cultivars had, the higher resistance the wheat cultivars had against *Pst*. In this study, all 82 wheat cultivars possessed a minimum of two or more *Yr* genes associated with stripe rust resistance. Additionally, the more *Yr* genes the wheat cultivars had, the higher resistance of the wheat cultivars was against *Pst* in the field. The results clarified the *Yr* gene distributions of the Xinjiang wheat cultivars and screened out the varieties with high resistance against *Pst*. The study provides a theoretical foundation for the diversity of wheat disease-resistance genes, rational distribution of disease-resistance genes, and breeding for disease resistance in Xinjiang.

## Figures and Tables

**Table 1:** The distribution of *Yr* genes in Xinjiang wheat cultivars.

Wheat Cultivar	*Yr5*	*Yr9*	*Yr10*	*Yr15*	*Yr17*	*Yr18*	*Yr26*	*Yr30*	*Yr41*	*Yr44*	*Yr50*	*Yr52*	*Yr78*	*Yr80*	*Yr81*	Number of *Yr* Genes against Stripe Rust
Xinchun No.2	+	−	−	+	+	−	+	+	+	+	+	−	−	+	−	9
Xinchun No.3	+	−	−	+	+	−	+	−	+	+	+	−	−	−	−	7
Xinchun No.5	+	−	−	+	+	−	+	+	+	+	+	−	+	+	−	10
Xinchun No.6	+	−	−	+	+	−	+	−	−	+	+	−	−	+	−	7
Xinchun No.7	+	−	−	+	+	−	+	−	+	+	+	−	−	+	−	8
Xinchun No.8	+	−	−	+	−	−	+	−	+	+	−	−	−	+	−	6
Xinchun No.9	+	−	−	+	+	−	−	−	+	−	−	−	−	+	−	5
Xinchun No.10	+	+	−	+	+	−	+	−	+	−	+	−	−	−	−	7
Xinchun No.11	+	−	−	+	+	−	+	+	+	+	+	−	+	+	−	10
Xinchun No.12	+	−	−	+	+	+	+	+	+	+	+	−	−	−	−	9
Xinchun No.13	+	−	−	+	+	−	+	+	+	+	+	−	+	+	−	10
Xinchun No.14	+	−	−	+	+	−	−	−	+	−	−	−	−	−	−	4
Xinchun No.15	+	−	−	+	+	−	+	+	+	−	+	−	−	−	−	7
Xinchun No.16	+	−	−	+	+	−	+	−	+	+	+	−	+	−	−	8
Xinchun No.17	+	−	−	+	+	−	+	−	+	+	+	−	−	−	−	7
Xinchun No.18	+	−	−	+	+	−	+	−	+	+	−	−	−	+	−	7
Xinchun No.19	+	−	−	+	+	−	+	+	−	+	+	−	−	−	−	7
Xinchun No.20	+	−	−	+	+	−	+	+	+	+	+	−	−	−	−	8
Xinchun No.21	+	+	−	+	+	−	−	+	+	−	+	−	−	−	−	7
Xinchun No.22	+	+	−	−	+	−	−	−	+	+	+	−	+	+	−	8
Xinchun No.23	+	−	−	+	+	−	+	−	+	+	+	−	−	−	−	7
Xinchun No.25	+	−	−	+	+	−	+	−	−	+	+	−	+	+	−	8
Xinchun No.26	+	−	−	+	+	−	+	−	+	−	+	−	+	+	−	8
Xinchun No.27	+	−	−	+	+	−	+	+	+	+	−	−	−	−	−	7
Xinchun No.28	+	−	−	+	+	+	+	−	+	+	+	−	+	+	−	10
Xinchun No.29	+	−	−	+	+	+	+	+	+	+	+	−	−	−	−	9
Xinchun No.30	+	−	−	+	+	−	+	−	+	−	+	−	+	+	−	8
Xinchun No.31	+	−	−	+	+	−	+	−	+	−	+	−	−	+	−	7
Xinchun No.32	+	+	−	+	+	−	−	−	+	+	+	−	+	+	−	9
Xinchun No.33	+	−	−	+	+	−	+	−	+	−	+	−	+	+	−	8
Xinchun No.34	+	−	−	+	+	−	+	−	−	+	+	−	+	−	−	7
Xinchun No.35	+	−	−	+	+	−	+	−	−	−	+	−	+	+	−	7
Xinchun No.36	+	−	−	+	+	−	+	+	+	+	+	−	−	−	−	8
Xinchun No.37	+	−	−	+	+	−	+	−	+	+	+	−	−	−	−	7
Xinchun No.38	+	−	−	+	+	+	+	−	+	−	+	−	+	+	−	9
Xinchun No.39	+	−	−	+	+	−	+	−	+	+	+	−	−	−	−	7
Xinchun No.40	+	−	−	+	+	−	+	−	−	+	−	−	−	+	−	6
Xinchun No.41	+	−	−	+	+	−	+	−	+	+	+	−	+	+	−	9
Xinchun No.43	+	−	−	+	+	+	+	+	−	−	−	−	−	−	−	6
Xinchun No.44	+	−	−	+	+	−	+	−	+	−	+	−	+	−	−	7
Xinchun No.45	+	−	−	+	+	−	+	−	−	+	+	−	−	−	−	6
Xindong No.1	−	−	−	+	+	−	+	+	+	−	+	−	−	+	−	7
Xindong No.2	−	−	−	+	+	−	+	+	+	−	+	−	−	+	−	7
Xindong No.3	−	−	−	−	+	−	+	+	+	−	+	−	−	−	−	5
Xindong No.4	−	−	−	+	+	−	+	+	+	−	+	−	−	−	−	6
Xindong No.5	+	−	−	+	+	−	+	−	+	+	+	+	+	+	−	10
Xindong No.6	+	−	+	+	+	+	+	−	−	−	−	−	−	−	−	6
Xindong No.7	−	−	−	−	+	−	+	−	+	−	+	−	−	+	−	5
Xindong No.9	−	+	−	+	+	−	−	−	+	+	+	−	−	−	−	6
Xindong No.10	+	−	−	+	+	−	+	−	+	+	+	−	−	−	−	7
Xindong No.11	−	−	−	+	+	−	+	+	+	+	+	−	−	+	−	8
Xindong No.12	+	−	−	+	+	−	−	−	+	−	+	−	−	+	−	6
Xindong No.13	+	−	+	+	+	−	+	+	−	−	−	−	−	−	−	6
Xindong No.14	+	−	−	−	+	−	+	+	−	+	+	+	+	+	−	9
Xindong No.15	+	+	−	+	+	−	+	+	+	+	+	−	+	+	−	11
Xindong No.17	+	−	−	−	+	−	+	−	+	+	+	−	−	−	−	6
Xindong No.18	+	−	−	−	+	−	+	−	+	+	+	−	−	−	−	6
Xindong No.19	−	−	−	+	+	−	+	−	+	−	+	+	−	−	−	6
Xindong No.20	−	−	−	+	+	−	+	−	−	−	+	−	+	+	−	6
Xindong No.22	−	−	−	−	+	−	+	−	+	−	+	−	+	+	−	6
Xindong No.23	−	−	−	+	+	−	+	−	+	−	+	−	−	+	−	6
Xindong No.24	+	−	−	−	+	−	+	+	+	+	+	−	+	−	−	8
Xindong No.25	+	−	−	−	+	−	+	+	−	+	+	−	−	−	−	6
Xindong No.26	−	−	−	−	+	−	+	+	+	+	+	−	+	+	−	8
Xindong No.28	+	+	−	−	+	−	+	−	+	−	+	−	−	−	−	6
Xindong No.29	+	+	−	+	+	−	−	−	+	−	+	−	+	+	−	8
Xindong No.32	−	−	−	−	+	−	+	−	+	−	+	−	−	−	−	4
Xindong No.33	−	−	−	−	+	−	+	+	+	−	+	−	−	+	−	6
Xindong No.35	+	−	−	−	+	−	+	+	+	+	+	−	−	−	−	7
Xindong No.41	−	−	−	−	+	−	+	+	+	−	+	−	−	+	−	6
Xindong No.46	+	−	−	−	+	−	−	+	−	+	−	−	+	−	−	5
Xindong No.49	−	−	−	−	+	−	−	−	−	−	−	−	+	−	−	2
Xindong No.50	+	−	−	−	+	−	−	+	+	+	+	−	+	−	−	7
Xindong No.51	+	−	−	−	+	−	+	+	+	+	+	−	+	−	−	8
Xindong No.52	−	−	−	+	+	−	+	+	−	−	+	−	−	−	−	5
Xindong No.53	+	−	+	+	+	+	+	+	+	+	+	−	−	−	−	10
Xindong No.57	−	−	−	−	+	−	+	−	+	−	+	−	+	−	−	5
Yili 034	+	+	−	+	+	−	−	−	+	−	+	−	−	−	−	6
Yili 053	−	−	−	+	+	−	+	−	−	−	+	−	−	−	−	4
Yili 060	−	+	−	+	+	−	−	−	−	−	−	−	−	+	−	4
Yili 070	+	−	−	−	+	−	+	−	+	+	−	−	−	−	−	5
Yili 086	+	+	−	+	+	−	+	+	+	+	+	−	−	−	−	9

+: The *Yr* gene was detected; −: the *Yr* gene was not detected.

**Table 2:** The pedigree of Xinjiang wheat cultivars.

Wheat Cultivar	Pedigree	Wheat Cultivar	Pedigree
Xinchun No.2	Cyrus × Qichun No.4	Xinchun No.37	49-5 × Yemao
Xinchun No.3	Cyrus × Qichun No.4	Xinchun No.38	Yuan 212 × 97-46-3
Xinchun No.5	Fan No.6 × 6038	Xinchun No.39	NS 64 × Xinchun No.8
Xinchun No.6	Zhong 7906 × Improved Xinchun No.2	Xinchun No.40	Xinchun No.6 × UC 1041
Xinchun No.7	Zhong 7906 × Improved Xinchun No.2	Xinchun No.41	H 101 × C 8501
Xinchun No.8	C 02 × 21-3	Xinchun No.43	90-33 × Xinchun No.6
Xinchun No.9	Introduced from northern Africa	Xinchun No.44	17-11 × Yn-76
Xinchun No.10	9-3-3 × Xinchun No.4	Xinchun No.45	2001-54 × Wuchun No.3
Xinchun No.11	86-7 × Xinchun No.2	Xindong No.1	(Ukraine 0246 × Aksu red winter wheat) × Aozi No.3
Xinchun No.12	8021 × 77-13	Xindong No.2	Reyimuxia × Helenhead
Xinchun No.13	Introduced from Canada	Xindong No.5	Bakhfuk × Beijing No.7
Xinchun No.14	Introduced from CIMMYT	Xindong No.7	(Reyimuxia × Helenhead) × Aozi No.3
Xinchun No.15	Fan 24 × 85307	Xindong No.13	Xindong No.3 × Ukraine 0246
Xinchun No.16	8-26 B × 93 Jian 29	Xindong No.15	Xindong No.2 × Zhongyin No.5
Xinchun No.17	Xinchun No.6 × NS64	Xindong No.18	N.S 11-33 × Xindong No.5
Xinchun No.18	Liberate No.4 × 919	Xindong No.19	Aphrodite × Hai 82-6
Xinchun No.19	Xinchun No.4 × Xinchun No.5	Xindong No.20	Introduced by Hebei Academy of Agricultural Sciences
Xinchun No.20	M 85-30 × Changchun No.6	Xindong No.23	Import from USA × 88-136
Xinchun No.21	NS-23-3 × Chun 946	Xindong No.24	9245 × Ji 6159
Xinchun No.22	Yong 1265 × Tal	Xindong No.25	Ji 885-443 × Ji 88-5282
Xinchun No.23	Introduced from CIMMYT	Xindong No.26	(Hongxuan 501 × Donald)F_1_ × (Hongxuan 501 × Cedar Cyrus)F_2_
Xinchun No.25	73/111 × Xinchun No.6	Xindong No.28	92/45 × Xindong No.20
Xinchun No.26	Xinchun No.6 × Xinchun No.9	Xindong No.29	PH82-2-2 × Luzhi 79-1
Xinchun No.27	21-4 × 91I82299	Xindong No.33	73-13-36 × 82-4009
Xinchun No.28	Introduced from CIMMYT	Xindong No.35	Jingnong 98 × Xindong No.18
Xinchun No.29	85-56 × 25-3	Xindong No.41	Shidong No.8 × (95-7-5-2 × Kuidong No.5)F_1_
Xinchun No.30	Xinchun No.9 × Xinchun No.6	Xindong No.46	Nongda 3338 × S 180
Xinchun No.31	12-25 × 96-5	Xindong No.49	(Pubing 4201/CHM 83.605//FC Dasui) F_5_ × Gaocheng 8901
Xinchun No.32	Yongliang No.11 × 97-18	Xindong No.50	8761 × Xindong No.17
Xinchun No.33	Xinchun No.6 × Xinchun No.9	Xindong No.51	(Gaocheng 8901 × Xindong No.18) F_1_ × Ji 5473
Xinchun No.34	88 (13)/5 × 44	Xindong No.52	Xindong No.17 × 95-7-13-2
Xinchun No.35	Ba 96-4870 × 93 Jian 29	Xindong No.53	01/2113 × Xindong No.18
Xinchun No.36	21-6 × Black wheat	Xindong No.57	81-8-2-1 × Xindong No.20

**Table 3:** Common primer sequences of *Yr* genes.

Gene	Primer Name	Primer Sequence	Reference
*Yr5*	Xwmc175	F: GCTCAGTCAAACCGCTACTTCT R: CACTACTCCAATCTATCGCCGT	[26]
	Xbarc167	F: AAAGGCCCATCAACATGCAAGTACC R: CGCAGTATTCTTAGTCCCTCAT	[27]
*Yr9*	AF1/AF4	F: GGAGACATCATGAAACATTTG R: CTGTTGTTGGGCAGAAAG	[28]
	H20	F: GTTGGGCAGAAAGGTCGACATC R: GTTGGAAGGGAGCTCGAGCTG	
*Yr10*	Yr10R1/ Yr10 F1	F: TTGGAATTGGCGACAAGCGT R: GTGATGATTACCCACTTCCTC	[29]
	Yr10R/ Yr10F	F: TCAAAGACATCAAGAGCCGC R: TGGCCTACATGAACTCTGGAT	[30]
*Yr15*	XBarc8	F: GCGGGAATCATGCATAGGAAAACAGAA R: GCGGGGGCGAAACATACACATAAAAACA	[31]
*Yr17*	VENTRIUPLN2	F: AGGGGCTACTGACCAAGGCT R: TGCAGCTACAGCAGTATGTACACAAAA	[32]
*Yr18*	Cslv34	F: CTGGTTAAGACTGGTGATGG R: TGCTTGCTATTGCTGAATAGT	[33]
	L34DINT13R2/ L34SPF	F: GGGAGCATTATTTTTTTCCATCATG R: ACTTTCCTGAAAATAATACAAGCA	
*Yr26*	Xgwm11	F: GGATAGTCAGACAATTCTTGTG R: GTGAATTGTGTCTTGTATGCTTCC	[34]
	Xgwm18	F: TGGCGCCATGATTGCATTATCTTC R: GGTGGCTGAAGAACCTTATTTAG	
*Yr41*	Xgwm410	F: GCTTGAGACCGGCACAGT R: CGAGACCTTGAGGGTCTAGA	[35]
	Xgwm374	F: ATAGTGTGTTGCATGCTGTGTG R: TCTAATTAGCGTTGGCTGCC	
*Yr44*	Xgwm501	F: GCTATCTCTGGCGCTAAAA R: TCCACAAACAAGTAGCGCC	[36]
*Yr50*	Xgwm540	F: TCTCGCTGTGAAATCCTATTT R: AGGCATGGATAGAGGGGC	[37]
	Xwmc47	F: GAAACAGGGTTAACCATGCCAA R: ATGGTGCTGCCAACAACATACA	

**Table 4:** KASP primer sequences of *Yr* genes.

Genes	Locations	Primer Sequence
*Yr30*	A/C	HEX: GGGCAATAGTGAGTCCCTTCAG FAM: AGGGCAATAGTGAGTCCCTTCAT Common: GCCCGCTCACCAACATCTACAAAAT
*Yr52*	C/T	HEX: GCCCACAACCTCTTTAGGCTGAT FAM: CCCACAACCTCTTTAGGCTGAC Common: GATTTTAACAGTGGGTGGGGTCAGTT
*Yr78*	C/A	HEX: CTAGACCCTACGACGTTAGCGA FAM: AGACCCTACGACGTTAGCGC Common: CTCACTTAAGTTAGTAGAGATCTCTTGTTT
*Yr80*	G/A	HEX: CATGTACAATGACTCCTCGACTAACA FAM: ATGTACAATGACTCCTCGACTAACG Common: ACCATCGAAAAATTGCCACAATGTGAGTT
*Yr81*	G/A	HEX: CCAAAGTAATTGGCAACAGGTTCA FAM: CCAAAGTAATTGGCAACAGGTTCG Common: TGTGGAGCGTGACAATGAGGAAGTT

## Data Availability

Data are contained within the article.

## References

[1] Dimmock J.P.R.E., Gooding M.J. (2002). The influence of foliar diseases, and their control by fungicides, on the protein concentration in wheat grain: A review. J. Agric. Sci..

[2] Liu S.J., Wang X.T., Zhang Y.Y., Jin Y.G., Xia Z.H., Xiang M.J., Huang S., Qiao L.Y., Zheng W.J., Zeng Q.D. (2022). Enhanced stripe rust resistance obtained by combining *Yr30* with a widely dispersed, consistent QTL on chromosome arm 4BL. Theor. Appl. Genet..

[3] Wan A.M., Chen X.M., He Z.J. (2007). Wheat stripe rust in China. Aust. J. Agric. Res..

[4] Huang L., Xiao X.Z., Liu B., Gao L., Gong G.S., Chen W.Q., Zhang M., Liu T.G. (2020). Identification of stripe rust resistance genes in common wheat cultivars from the Huang-Huai-Hai region of China. Plant Dis..

[5] Li Z.Q., Zeng S.M. (2002). Chinese Wheat Rust.

[6] Liang S.X., Wu S.X., Zhang S.Q., Zheng M.Y., Chen X.X., Chen J., Liu Q. (2023). A GIS based study on wheat stripe rust oversummering zoning in Xinjiang. China Plant Prot..

[7] Liu Q., Sun T.T., Wen X.J., Zeng M.H., Chen J. (2023). Detecting the minimum limit on wheat stripe rust in the latent period using proximal remote sensing coupled with duplex real-time PCR and machine learning. Plants.

[8] Chen X.M. (2013). Review article: High-temperature adult-plant resistance, key for sustainable control of stripe rust. Am. J. Plant Sci..

[9] Li J.B., Dundas I., Dong C.M., Li G.R., Trethowan R., Yang Z.J., Hoxha S., Zhang P. (2020). Identification and characterization of a new stripe rust resistance gene *Yr83* on rye chromosome 6R in wheat. Theor. Appl. Genet..

[10] Rasheed A., Wen W.E., Gao F.M., Zhai S.N., Jin H., Liu J.D., Guo Q., Zhang Y.J., Dreisigacker S., Xia X.C. (2016). Development and validation of KASP assays for genes underpinning key economic traits in bread wheat. Theor. Appl. Genet..

[11] Liu Y.N., He Z.H., Appels R., Xia X.C. (2012). Functional markers in wheat: Current status and future prospects. Theor. Appl. Genet..

[12] Khalid M., Afzal F., Gul A., Amir R., Subhani A., Ahmed Z., Zahid M., Xia X.C., Rasheed A., He Z.H. (2019). Molecular characterization of 87 functional genes in wheat diversity panel and their association with phenotypes under well-watered and water-limited conditions. Front. Plant Sci..

[13] Feng J.Y., Wang M., See D.R., Chao S.M., Zheng Y.L., Chen X.M. (2018). Characterization of novel gene *Yr79* and four additional quantitative trait loci for all-stage and high-temperature adult-plant resistance to stripe rust in spring wheat PI 182103. Phytopathology.

[14] Hu Y.S., Zhang Y., Lu K.X., Li Y.X., Yan B.J., Chen X.M., Shang H.S., Hu X.P. (2023). Identification of high-temperature resistance to stripe rust and molecular detection of *Yr* genes in Chinese core collections of common wheat. Crop Prot..

[15] Ma Z.Y. (2022). Evaluation of Resistance to Stripe Rust and Detection of Resistance Genes of Winter Wheat Varieties in Xinjiang. Master’s Thesis.

[16] Liu L., Wang M.N., Zhang Z.W., See D.R., Chen X.M. (2020). Identification of stripe rust resistance loci in US spring wheat cultivars and breeding lines using genome-wide association mapping and *Yr* gene markers. Plant Dis..

[17] Liu L., Wang M.N., Feng J.Y., See D.R., Chao S.M., Chen X.M. (2018). Combination of all-stage and high-temperature adult-plant resistance QTL confers high-level, durable resistance to stripe rust in winter wheat cultivar Madsen. Theor. Appl. Genet..

[18] Han D.J., Kang Z.S. (2018). Current status and future strategy in breeding wheat for resistance to stripe rust in China. Plant Prot..

[19] Krattinger S.G., Lagudah E.S., Spielmeyer W., Singh R.P., Huerta-Espino J., McFadden H., Bossolini E., Selter L.L., Keller B. (2009). A putative ABC transporter confers durable resistance to multiple fungal pathogens in wheat. Science.

[20] Leng P.F., Lübberstedt T., Xu M.L. (2017). Genomics-assisted breeding-A revolutionary strategy for crop improvement. J. Integr. Agric..

[21] Yang Q.Q., Tang J.Q., Zhang C.Q., Gao J.P., Liu Q.Q. (2017). Application and prospect of KASP marker technology in main crops. Biot. Bull..

[22] Zhan H.X., Wang Y.L., Zhang D., Du C.H., Zhang X.J., Liu X.L., Wang G.Y., Zhang S.S. (2021). RNA-seq bulked segregant analysis combined with KASP genotyping rapidly identified *PmCH7087* as responsible for powdery mildew resistance in wheat. Plant Genome.

[23] Wu J.H., Zeng Q.D., Wang Q.L., Liu S.J., Yu S.Z., Mu J.M., Huang S., Sela H., Distelfeld A., Huang L.L. (2018). SNP-based pool genotyping and haplotype analysis accelerate fine-mapping of the wheat genomic region containing stripe rust resistance gene *Yr26*. Theor. Appl. Genet..

[24] Cheng P., Xu L.S., Wang M.N., See D.R., Chen X.M. (2014). Molecular mapping of genes *Yr64* and *Yr65* for stripe rust resistance in hexaploid derivatives of durum wheat accessions PI 331260 and PI 480016. Theor. Appl. Genet..

[25] Riede C.R., Anderson J.A. (1996). Linkage of RFLP markers to an aluminum tolerance gene in wheat. Crop Sci..

[26] Murphy L.R., Santra D., Kidwell K.B., Yan G.P., Chen X.M., Campbell G.K. (2009). Linkage maps of wheat stripe rust resistance genes *Yr5* and *Yr15* for use in marker-assisted selection. Crop Sci..

[27] Somers D.J., Isaac P., Edwards K. (2004). A high-density microsatellite consensus map for bread wheat (*Triticum aestivum* L.). Theor. Appl. Genet..

[28] Francis H.A., Leitch A.R., Koebner R.M. (1995). Conversion of a RAPD-generated PCR product, containing a novel dispersed repetitive element, into a fast and robust assay for the presence of rye chromatin in wheat. Theor. Appl. Genet..

[29] Singh R., Datta D., Priyamvada, Singh S., Tiwari R. (2009). A diagnostic PCR based assay for stripe rust resistance gene *Yr10* in wheat. Acta. Phytopathol. Entomol. Hung..

[30] Wang L.F., Ma J.X., Zhou R.H., Wang X.M., Jia J.Z. (2002). Molecular tagging of the yellow rust resistance gene *Yr10* in common wheat, P.I.178383 (*Triticum aestivum* L.). Euphytica.

[31] Peng J.H., Fahima T., Roder M.S., Huang Q.Y., Dahan A., Li Y.C., Grama A., Nevo E. (2000). High-density molecular map of chromosome region harboring stripe-rust resistance genes *YrH52* and *Yr15* derived from wild emmer wheat, *Triticum dicoccoides*. Genetica.

[32] Wu S.S. (2021). Evaluation of Stripe Rust Resistance of 270 Bred Wheat Cultivaries (Lines) from the Main Wheat Region in China. Master’s Thesis.

[33] Lagudah E.S., Krattinger S.G., Herrera-Foessel S., Singh R.P., Huerta-Espino J., Spielmeyer W., Brown-Guedira G., Selter L.L., Keller B. (2009). Gene-specific markers for the wheat gene *Lr34*/*Yr18*/*Pm38* which confers resistance to multiple fungal pathogens. Theor. Appl. Genet..

[34] Ma J.X., Zhou R.H., Dong Y.S., Wang L.F., Wang X.M., Jia J.Z. (2001). Molecular mapping and detection of the yellow rust resistance gene *Yr26* in wheat transferred from *Triticum turgidum* L. using microsatellite markers. Euphytica.

[35] Luo P.G., Hu X.Y., Ren Z.L., Zhang H.Y., Shu K., Yang Z.J. (2008). Allelic analysis of stripe rust resistance genes on wheat chromosome 2BS. Genome.

[36] Wan A., Chen X.M. (2014). Virulence characterization of *Puccinia striiformis* f. sp. tritici using a new set of Yr single-gene line differentials in the United States in 2010. Plant Dis..

[37] Liu J., Chang Z.J., Zhang X.J., Yang Z.J., Li X., Jia J.Q., Zhan H.X., Guo H.J., Wang J.M. (2013). Putative *Thinopyrum intermedium*-derived stripe rust resistance gene *Yr50* maps on wheat chromosome arm 4BL. Theor. Appl. Genet..

[38] Fang T.H., Zhang M., Ma C.H., Zheng X.C., Tan W.J., Tian R., Yan Q., Zhou X.L., Li X., Yang S.Z. (2022). Application of *Yr52* gene in wheat improvement for stripe rust resistance. Sci. Agric. Sin..

[39] Dong Z.Z., Hegarty J.M., Zhang J.L., Zhang W.J., Chao S.M., Chen X.M., Zhou Y.H., Dubcovsky J. (2017). Validation and characterization of a QTL for adult plant resistance to stripe rust on wheat chromosome arm 6BS (*Yr78*). Theor. Appl. Genet..

[40] Nsabiyera V., Bariana H.S., Qureshi N., Wong D., Hayden M.J., Bansal U.K. (2018). Characterisation and mapping of adult plant stripe rust resistance in wheat accession Aus27284. Theor. Appl. Genet..

[41] Gessese M., Bariana H., Wong D., Hayden M., Bansal U. (2019). Molecular mapping of stripe rust resistance gene *Yr81* in a common wheat landrace Aus27430. Plant Dis..

[42] Makhoul M., Rambla C., Voss-Fels K.P., Hickey L.T., Snowdon R.J., Obermeier C. (2020). Overcoming polyploidy pitfalls: A user guide for effective SNP conversion into KASP markers in wheat. Theor. Appl. Genet..

[43] Huang S., Wu J.H., Wang X.T., Mu J.M., Han D.J. (2019). A utilization of the genome-wide wheat 55K array for genetic analysis of stripe rust resistance in common wheat line P9936. Phytopathology.

[44] Liu X., Lei M.L., Wang Y.Z., Wang Y.N., Huang R., Mu Z.X. (2022). Detection of quality-related genes in the wheat landrace in Shanxi province by KASP markers. J. Plant Gene. Resour..

